# Fluorescence *in situ* hybridization (FISH) Protocol in Human Sperm

**DOI:** 10.3791/1405

**Published:** 2009-09-01

**Authors:** Zaida Sarrate, Ester Anton

**Affiliations:** Unitat de Biologia Cel·lular (Facultat de Biociències),

## Abstract

Aneuploidies are the most frequent chromosomal abnormalities in humans. Most of these abnormalities result from meiotic errors during the gametogenic process in the parents. In human males, these errors can lead to the production of spermatozoa with numerical chromosome abnormalities which represent an increased risk of transmitting these anomalies to the offspring.

For this reason, the technique of fluorescence *in situ* hybridization (FISH) on sperm nuclei has become a protocol widely incorporated in the context of clinical diagnosis. This practice provides an estimate of the frequencies of numerical chromosome abnormalities in the gametes of the patients that seek for genetic reproductive advice.

To date, the chromosomes most frequently included in sperm FISH analysis are chromosomes X, Y, 13, 18 and 21.

This video-article describes, step by step, how to process and fix a human semen sample, how to decondense and denature the sperm chromatin, how to proceed to obtain sperm FISH preparations, and how to visualize the results at the microscope. Special remarks of the most relevant steps are given to achieve the best results.

**Figure:**
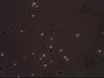


## Protocol

### I. Sample processing and cell fixation

1. Leave the semen sample in sterile containers at room temperature for 20 minutes until liquefaction.
2. Transfer the sample to a centrifuge tube and spin it at 1000g for 5 minutes.
3. Gently remove and discard the supernatant using a Pasteur pipette.
4. Add hypotonic solution (KCl, 0.075M) pre-heated at 37°C, drop by drop, while mixing on a vortex to obtain a final volume of 10 ml.
5. Place the tube in a water bath at 37°C for 30 minutes.
6. Centrifuge at 1000g for 5 minutes. Carefully discard the supernatant by decantation, without disturbing the pellet.
7. After resuspending the pellet, add freshly prepared Carnoy’s fixative (3:1 methanol:acetic acid) drop by drop while mixing on a vortex to obtain a final volume of 8 ml.
8. Repeat points 6 and 7 as many times as necessary to obtain a white pellet.
9. Add freshly prepared methanol:acetic acid (3:1) drop by drop to adjust the final volume to the cellular concentration required for obtaining good cell spreading (in those cases with a small pellet, few drops will be enough whereas samples with a high amount of sperm recovered, the final volume can reach 1-2 ml). A test slide can be made by dropping the resuspended sample onto an ungreased slide and examining it under a phase contrast microscope. This will let to check the cellular dispersion obtained and would allow correcting it, if it is necessary, in further slides.
10. To make the extensions, drop from a minimum height of 40 cm two or three drops of cell suspension into the center of unfrosted slides (previously stored in methanol at -20°C to degrease them). To enable the location of the sample throughout the protocol, mark the area containing the sperm extension on the opposite side of the slide using a diamond pencil.
11. Store the slides at -20°C for at least 24 hours. *Note: The addition of methanol:acetic acid (3:1) drop by drop while mixing is a very important step to avoid the formation of sperm aggregates.*

### II. Decondensation

1. The slides stored at -20°C must be defrosted to continue with the protocol (leave them at room temperature).
2. Place the slide in two consecutive coplin jars with 2x saline-sodium citrate solution (2xSSC) for 3 minutes each.
3. Transfer the slide through a series of ethanol washes for 2 minutes in each coplin jar. Start with 70% ethanol, follow by 90% and finish with 100%. Dry out the slide leaving it at room temperature.
4. Incubate the slide in dithiothreitol solution (1,4-dithiothreitol 5mM, 1% Triton X-100, 2-Amino-2-(hydroxymethyl)-1,3-propanediol 50mM) at 37°C in the incubator to decondense the chromatin. This product acts by breaking the disulfide bridges of the protamines that coil the DNA in the spermatozoa nucleus. This is a very important step because the DNA in the sperm nucleus is highly compacted. The incubation time of the slides in dithiothreitol solution (DTT) must be adjusted according to the sample's reactivity to this product. Usually, this time is around 8 minutes, although it can vary from 2 to 15 minutes.
5. Immediately, transfer the slide to two consecutive coplin jars with 2xSSC for 3 minutes in each coplin.
6. Continue by placing the slide through a series of ethanol washes for 2 minutes in each coplin jar (70%, 90% and 100% ethanol). Let the slide to dry out at room temperature. *Note: Excessive exposure to DTT would result in disperse FISH signals at the end of the procedure, whereas a too short exposure would result in a lack of some signals.*

### III. Hybridization

1. Denature the sperm DNA by incubating the slide in a coplin jar with formamide solution (70% Formamide/2xSSC) at 73°C for 5 minutes.
2. Transfer the slide through ethanol solutions for 1 minute per coplin jar (start with 70% ethanol, 85% and finish with 100%). Leave the slide to dry out at room temperature.
3. Add directly 5  l of the corresponding ready-to-use probe mixture to a 15x15 cover slip (AneuVysion Assay Multi-color Probe Panel: CEP 18/X/Y or LSI 13/21).
4. As it is shown in the video, carefully place the slide onto the cover slip to put together the target region and the probe mixture. Seal it with rubber cement.
5. Place the slide into a pre-warmed 37°C hybridization chamber (HYBrite™) and incubate it at 37°C for 6-24 hours. *Note: Whereas the denaturation of the sperm sample is mandatory, the necessity of denaturing the probes is determined by the manufacturer. The probe mixtures used in this protocol are ready to use and, in this case, probe denaturation is not required.The volume of the probe mixture will vary according to the size of the hybridized area.*

### IV. Washes post-hybridization

1. Take out the slide from the hybridization chamber.
2. Remove the rubber cement and carefully pull out the cover slip sliding gently to the side.
3. To eliminate the unspecific hybridization signals place the slide for 2 minutes in a coplin jar with 0.4xSSC/0.3%NP-40 pre-warmed at 73°C in a water bath.
4. Transfer the slide to a 2xSSC/0.1%NP-40 wash solution at room temperature for 1 minute. Let the slide to dry out at room temperature.
5. Add a counterstaining product to the target region (8  l for an 18x18 cover slip). The most common product used is 4’,6-diamidino-2-phenylindole (DAPI, Vysis Inc.).
6. As it is shown in the video, cover the hybridized region with a cover slip (to preserve the hybridization the cover slip can be sealed with nail varnish).
7. Store hybridized slides at -20°C in the dark until their prospect analysis. Under these conditions the slides can be stored for up to 12 months without significant lost in fluorescence signal intensity. *Note: A higher temperature or an excessive wash time can result in the elimination of some signals, whereas the opposite would not wash out unspecific probe hybridizations.The volume of the counterstaining product added will vary according to the size of the hybridization area to cover. *

### V. Visualization

The preparations can be evaluated under an epifluorescence microscope equipped with a triple-band filter for DAPI/Texas Red/FITC and single-band filters for Aqua, FITC and Texas Red. Standard assessment criteria must be followed for the correct evaluation of the sperm nuclei ^1^.

The preparation hybridized with probes for chromosomes X, Y and 18 should display signals for these three chromosomes. Every normal spermatozoa must show one blue signal (corresponding to the chromosome 18), and a green signal (X-bearing spermatozoa) or a red signal (Y-bearing spermatozoa).

In the preparation hybridized with probes for chromosomes 13 and 21, a green signal for chromosome 13 and a red signal for chromosome 21 should be distinguished in every normal spermatozoa.

## Discussion

This protocol describes how to process human semen samples for obtaining sperm FISH preparations. Using this protocol it is possible to analyze chromosomal abnormalities in male gametes. Spermatozoa aneuploidy screening has applications either in the context of basic research and in the reproductive advice given to infertile males. Although in clinical diagnosis the most widely studied chromosomes are X, Y, 13, 18 and 21, other commercial probes for a wide range of chromosomes and loci are available and can also be used in these experiments.
